# Malaria vaccination: hurdles to reach high-risk children

**DOI:** 10.1186/s12916-024-03321-2

**Published:** 2024-03-13

**Authors:** Floriano Amimo

**Affiliations:** Faculty of Medicine, Eduardo Mondlane University, Maputo, Mozambique

**Keywords:** Childhood immunization, Health system, Malaria, Number needed to vaccinate, Plasmodium falciparum, R21/Matrix-M, RTS,S/AS01

## Abstract

Ensuring that malaria vaccines deliver maximum public health impact is non-trivial. Drawing on current research, this article examines hurdles that malaria immunization may face to reach high-risk children and explores the policy implications. The analysis finds health system related risks with the potential to reduce the ability of malaria vaccines to provide equitable protection. Deployment of effective frameworks to tackle these risks so as to strengthen within-country equity and progress tracking should be entangled with the deployment of the vaccines. To capture more comprehensively disease- and system-related risks to child health and survival, vaccine allocation criteria should expand their data and indicator breadth. Factoring molecular, clinical, and epidemiological features of antimalarial drug resistance into vaccine allocation frameworks is critical to effectively reflect current and future risks to malaria control interventions. It is proposed that approximately 6–15 children would need to be vaccinated to prevent a malaria adverse outcome. Vaccine purchasing and delivery costs may overwhelm endemic countries’ health systems given the sizeable number needed to vaccinate, the population of at-risk children, and limited government financing of the health sector. Innovations in health financing are pivotal to ensuring the cost-effectiveness and sustainability of immunization programs aiming to attain and maintain universal and equitable protection.

**Supplementary Information:**

The online version contains supplementary material available at 10.1186/s12916-024-03321-2.

## Background

Malaria remains a major cause of death and suffering in Africa despite decades of efforts in the context of Millennium Development Goals (MDGs) and Sustainable Development Goals (SDGs). To strengthen disease control, the World Health Organization (WHO) recommends the widespread use in endemic countries of the RTS,S/AS01 (RTS,S), approved in 2021, and R21/Matrix-M (R21), approved in 2023, through a 4-dose immunization schedule in children aged ≥ 5 months [1, 2]. The addition of vaccines to the malaria prevention toolkit is a game-changing contribution to improving child survival and health on the continent. This is even more significant in the current context given the multitude of complex threats to progress towards global goals, including rising antimalarial drug resistance (AMDR) and frequent and more intense disasters and other shocks, which disrupt disease control. The Global Technical Strategy for Malaria (GTSM) 2016–2030, adopted by the World Health Assembly in 2015 and updated in 2021, includes the milestone and target to eliminate malaria in at least 20 and 35 countries by 2025 and 2030, respectively, anchored in Pillar 2 of its Strategic Framework and SDG Target 3.3 [3]. However, in the SDGs era, since 2015, only 13 countries globally have been certified malaria-free by the WHO [4]. And only one of these countries is in sub-Saharan Africa (SSA) (Cape Verde [certified in 2024]) and two are in the WHO African Region (AFR) (Cape Verde and Algeria [2019]), the regions most affected by the disease.

Malaria vaccines—the first (RTS,S) with a moderate vaccine efficacy (VE, range interval: 29% [95% confidence interval (CI): 6–46] against severe malaria; 39% [95% CI: 34–43] against clinical malaria [5]) and the second (R21) with improved VE (range interval: 67% [95% CI: 59–73] against multiple clinical malaria episodes; 75% [95% CI: 71–79] for time for first clinical malaria [6])—both a realization of decades of research and development (R&D), have renewed expectations to put progress in disease control back on-track towards global targets. Nevertheless, despite these potentials, additional investment is needed, not only in R&D of more effective and cost-effective vaccines capable of providing more lasting protection and a better safety profile with fewer doses but also in innovative delivery strategies, to ensure the realization of impact, equity, and sustainability. It is necessary to prevent malaria immunization from facing the same chronic difficulties that have undermined the control and elimination of other vaccine-preventable diseases in Africa.

This article, the first of its scope, explores in depth risks that may hinder attainment of universal malaria immunization. It focuses on health system related risks with the potential to reduce the ability of malaria vaccines to provide equitable protection across and within countries. The analysis draws on, and aims to extend and explore the policy implications of, current research. It is performed around three interconnected domains. In each domain, each of the identified related risks is examined in depth, addressing their causes, relationships, implications for malaria vaccination, and avenues to mitigate their impact and maximize the public health impact of the vaccines. The first domain addresses how *Limited government health financing reduces the value of vaccines* and explores the implications for malaria control and approaches to address the related risks. Here, current research is given a new perspective, so as to illustrate the risks as well as provide the rationale of the recommendations. The subsequent domain addresses directly the rationale and avenues to *Strengthening vaccine allocation frameworks to ensure equitable protection* of high-risk children against the disease. The last domain illustrates why and how *Tackling core issues to maximize the impact of malaria immunization* is a cornerstone, not an appendage, if malaria vaccines are to become a transforming force towards disease elimination. All data used and/or calculations conducted to illustrate a point in each domain are explained straightforwardly.

## Main text

### Limited government health financing reduces the value of vaccines

**Fig. 1:**
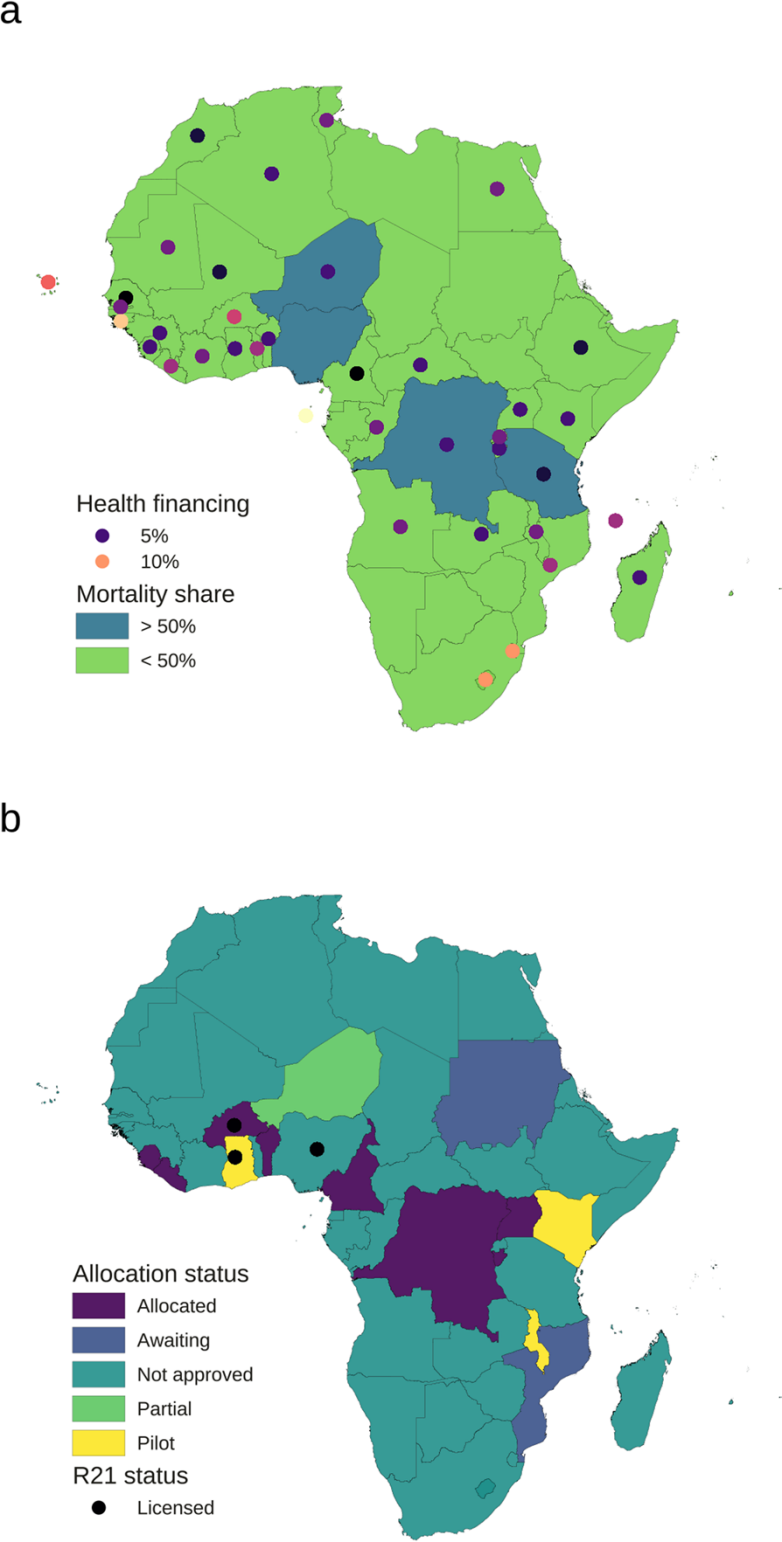
Geospatial distribution of vaccine allocation, health financing, and malaria mortality in Africa. **a** Distribution of government health financing and malaria mortality share. The color of each dot is proportional to health financing in percentage. Health financing denotes the average health financing in 2019–2021 measured as geometric mean of central government health spending as a share of general government expenditure. The color of the surface of each country represents whether such country was part or not of the four African countries that in 2021–2022 accounted for just over half of global malaria deaths. Most malaria-endemic countries with high disease burden do not comply with the Abuja Declaration of 2001 to allocate ≥ 15% of their annual budget to improve the health sector. **b** Malaria vaccine allocation status. Vaccine allocation categories shown with color for each country are as follows: Allocated, countries allocated vaccine for phase 1 areas; Awaiting, countries approved for Gavi support for malaria vaccine allocation but currently without supply; Not approved, countries not approved for Gavi support for malaria vaccine allocation; Partial, countries allocated partial supply awaiting further supply; Pilot, countries allocated vaccines to continue in the WHO-coordinated vaccine piloting program (i.e., MVIP countries). These were determined based on proxy measures of malaria disease burden and of child risk of death. R21 status denotes country-level regulatory status of R21 vaccine. The presence of a black dot in each country indicates that R21 has been licensed in the country. Gavi-eligible countries Burkina Faso (allocated), Ghana (pilot), and Nigeria (not approved) have licensed R21. Nigeria and Tanzania, although being among the four countries that the World Malaria Reports 2022–2023 identified as having accounted for just over half of all malaria deaths in 2021–2022, were not approved for Gavi support for priority allocation for 2023–2025. Data sources: [1, 6, 8–10]

Most African countries have limited public health financing to purchase malaria vaccines at market value in the quantities needed to cover their eligible at-risk children. This is because most of these countries do not comply with the Abuja Declaration of 2001 to allocate ≥ 15% of their annual budget to improve the health sector [7, 8] (Fig. 1a), that is, African governments do not invest adequately in the health of their populations due to misallocation of resources, not necessarily absolute scarcity. The consequences include reducing the impact of global health efforts and assets, such as malaria vaccines, on population health on the continent, thereby reducing disability-adjusted life years (DALYs) and productivity of their populations.

Theoretical and empirical considerations linking vaccine needs, vaccine and disease characteristics, and vaccine delivery difficulties are critical to illustrating how limited government health financing lessens the value of vaccines to protect health. Given the VE of the currently approved vaccines (see range interval of the VEs of both vaccines with associated uncertainty in the “Background” section) and the malaria incidence among unvaccinated (IU) in the AFR (WHO estimate of case incidence in 2022: 222.6 per 1000 population at-risk), then the number needed to vaccinate (NNV) can be calculated [5, 6, 9, 11]: $NNV={\left(IU\times VE\right)}^{-1}$. Applying this equation to the data suggests that between 6 (95% CI: 6–6) and 15 (95% CI: 10–75) children would need to be vaccinated to prevent a malaria adverse outcome. This is an overall NNV, based on the malaria incidence in the general population at-risk (i.e., residing in malaria-endemic areas) in the AFR, which is typically lower than in high-risk unvaccinated children eligible for malaria vaccination (e.g., 75.7–74.5%, that is, ¾ of global malaria deaths in 2021–2022 occurred in children younger than 5 years (U5) in the AFR, respectively [9, 10]). To determine the number of vaccines needed to prevent a malaria adverse outcome (VN), the vaccine wastage (VW) rate needs to be factored in as follows:

$$VN=DF\times \frac{1+VW}{IU\times VE},$$

where DF denotes the number of indicated doses, currently set at four for full-immunization. The propensity of VW increases as (a) the geographical distance from the most advantaged urban center (MA) (G_D_), (b) the developmental distance from MA (D_D_), and (c) institutional mistrust (I_M_) increase [12–14], that is, subnationally, $VW\sim f\left({G}_{D},{D}_{D},{I}_{M}\right)$. Thus, in case only a national average of VW rate is available, then district- or municipality-level VW rates need to be corrected accordingly depending on their G_D_, D_D_, and I_M_. It can be seen that VN can ensure that relevant drivers that modulate the conversion of vaccines acquired by the government into health protection attained by the population are factored in. It is thus valuable for immunization policy-making and priority-setting and should be used routinely with subnational data, including weighted VE effectively being deployed.

Vaccine purchasing costs are currently estimated at US$2–$4 and $10.07 per dose for R21 and RTS,S, respectively [2, 15]. Applying VN to the costs, then the financial investment needed to purchase vaccines to attain a target health impact that effectively accounts for, not only the clinical and epidemiological features of the vaccines and disease, but also the expected logistical constraints in the field during implementation, can be estimated, that is, the health value for money invested in vaccine purchasing and vice-versa can be ascertained subnationally. Using this quantity and given the size of the population of eligible at-risk children and the substandard government financing of the health sector in most of the continent [7, 8] (Fig. 1a), it can be shown that the resulting purchasing costs may easily overwhelm most endemic countries’ health systems. Therefore, most countries may have to rely upon the support of development partners. This risks reducing the value of the vaccines as these partners may not have the financial robustness to cover the needs of many endemic countries fully and sustainably.

Gavi, the Vaccine Alliance (Gavi) uses World Bank (WB) data on gross national income per capita (GNI p.c.) to determine country eligibility for support, transition phase, and co-financing share [16]. Due to the high cost of RTS,S, it adopted custom co-financing rules for malaria vaccines [15]. Based on these eligibility, transition, and co-financing rules, Gavi-eligible countries are placed in one of the following phases: (i) “initial self-financing phase” (eligibility: 3-year average or latest GNI p.c. ≤ WB’s low-income threshold; general co-financing: $0.20 per dose; malaria vaccine co-financing: same as general co-financing); (ii) “preparatory transition phase” (3-year average or latest GNI p.c. > WB’s low-income threshold; increase of 15% per year [in the percentage of vaccine cost co-financed]; $0.20 per dose in the first year and increase of 15% per year subsequently); and (iii) “accelerated transition phase” (3-year average and latest GNI p.c. > Gavi eligibility threshold; ≥ 35% of vaccine cost; 20% of the price in the first year and increase of 10% per year subsequently) [17, 18].

Due to limited supply, only 12 eligible countries out of the 47 WHO member states in the AFR have to date been approved for Gavi support for priority vaccine allocation for 2023–2025 of 18 million doses of RTS,S, including those in the Malaria Vaccine Implementation Programme (MVIP) [1, 10] (Fig. 1b). Out of these 12 approved countries, eight are in the “initial self-financing phase” (those ineligible for other transition phases), two are in the “preparatory transition phase” (Benin and Cameroon), and two are in the “accelerated transition phase” (Ghana and Kenya) [16]. The United Nations estimate a total population of U5 in SSA in 2023–2025 at 184–189 million, respectively [19]. Thus, based on currently available supplies, doses to vaccinate against malaria a total of 4.5 million children, that is, approximately 2.4% of the total population of U5 at-risk of malaria on the continent, are currently available for 2023–2025, without accounting for VW (unlike VN, derivation of the maximum fraction that can be vaccinated does not require IU and VE but the number of available doses, DF, and target population size). The recent approval of the more cost-effective R21 vaccine and the expected reduction of costs of the vaccines over time could improve the supply, availability, and accessibility of the vaccines to these and more endemic countries over time. R21 has been licensed for use in several countries, including Gavi-approved countries Burkina Faso and Ghana, as well as Nigeria [6]. Nevertheless, unless there is a transformational change in health financing models in endemic countries, then the limited supply, availability, and accessibility are likely to continue in various countries. That could significantly hinder attaining and maintaining full coverage in high-risk children.

Delivery costs are a significant bottleneck that may also contribute to reducing the value of malaria vaccines to provide equitable protection for children. This is directly related to the limited financial capacity of African health systems to convert vaccines into vaccinations. For instance, using mixed delivery (i.e., mass campaign plus routine Expanded Program on Immunization (EPI))—the most likely approach to be used by many countries—then an additional cost of $0.70–$1.37 per dose would be needed to administer a dose of malaria vaccine in settings with seasonal transmission [20], excluding vaccine purchasing costs. This corresponds to approximately $2.80–$5.48 for each child that is fully immunized against malaria with four doses. Assuming delivery costs of the vaccines are not significantly different from each other, also excluding vaccine purchasing costs, and applying these quantities to the population of U5 across SSA, then approximate costs of malaria vaccine delivery among U5 can be estimated. If Universal Health Coverage in the context of the SDG Target 3.8 and GTSM 2016–2030 Pillar 1 is to be achieved [3], then 100% of eligible children would need to be vaccinated against malaria to ensure access for all by 2030. This implies that roughly $386.40–$776.79 and $515.20–$1,035.72 million in delivery costs would be needed in the first year and yearly subsequently, respectively, to attain and maintain universal coverage of malaria vaccines among U5. The share of infants aged < 5 months currently ineligible for malaria immunization, heterogeneity in endemicity, VW, and other potential sources of variability are not accounted for in the derivation of these cost estimates. This approach should be applied nationally and subnationally using data with higher granularity to attain higher resolution. Overall, these estimates propose that non-vaccine costs to deliver the vaccines might not be trivial as well, particularly given the substandard government health financing in most of the continent [7, 8], and could become a significant hurdle that may reduce the public health impact of the vaccines (Fig. 1a).

Thus, while the existence of effective malaria vaccines is certainly of critical value, it is nevertheless not sufficient. Effective planning, funding, and coordination are crucial. These require a robust, well-funded health system. Sustainable health financing models are paramount. Otherwise, the malaria vaccines, although effective, might not deliver their potential public health impact.

### Strengthening vaccine allocation frameworks to ensure equitable protection

How can the limited supply doses be distributed to ensure that children most-at-risk of malaria-related adverse health outcomes are prioritized to maximize effectiveness, equity, and impact, not only across but also within countries? The WHO has created a framework to guide the selection of countries with high-risk areas to benefit from Gavi support to offset vaccine acquisition costs and ensure access to supplies by priority countries. Based on the WHO framework, non-MVIP countries that applied for Gavi support classified their districts into five categories based on the risk of malaria-related adverse health outcomes and, therefore, the need for additional protection and prioritization level for roll-out [21]. The eligibility criteria outlined by the WHO framework and adopted for the selection of Gavi-eligible countries for priority malaria vaccine allocation are as follows: (i) moderate and high malaria disease burden (using transmission in 2019—measured through *Plasmodium falciparum* (*P. falciparum*) parasite prevalence rate in children aged 2–10 years (*Pf*PR_2–10_) or malaria incidence rates—as a proxy measure) and (ii) high risk of child death (using all-cause under-5 mortality rate (U5MR) in 2015 as a proxy measure), both at district-level [1]. There are several limitations in this vaccine prioritization index, which might reduce its capacity to capture current and future malaria-related risks to child health and survival.

The Gavi-eligible countries approved for priority allocation were selected based on data from a single year for each component indicator. These data are about 4–6 and 8–10 years old for the target vaccination period, respectively. The data on *Pf*PR_2–10_ and U5MR used by the WHO framework are Malaria Atlas Project and Institute for Health Metrics and Evaluation estimates [21] modeled using prior data on multiple predictor variables, respectively. However, each year-specific epidemiological estimate aims to approximate the epidemiological quantity in that year. It cannot on its own summarize future trends of the quantity unless the quantity is known to be constant over time. And *Pf*PR_2–10_ and U5MR change over time. By not using recent data, an average, and/or a temporal trend, the accuracy of the vaccine needs assessment might be reduced. The use of single-year data creates vulnerability of the composite index to data quality. Expanding temporal coverage of the data used could improve the robustness and impact of the vaccine allocation prioritization index.

Antimicrobial resistance, the silent pandemic, was not accounted for as well, notwithstanding that it is an indispensable indicator of the current and future vulnerability of current antimalarial interventions. Resistance to (i) sulfadoxine-pyrimethamine, used for preventive treatment in children and pregnant women, and to (ii) pyrethroid insecticides, used for vector control (insecticide-treated bed nets and indoor residual spraying), is a pressing concern for malaria control in Africa. Although artemisinin-based combination therapy medicines appear to be largely effective to date, there are increasing reports of mutations in the *P. falciparum kelch13* propeller domain on the continent [22]. This is consequential for needs assessment and vaccine allocation. Geographies with the same baseline *Pf*PR_2–10_, malaria incidence, and/or U5MR but different levels and trends of AMDR represent different malaria control landscapes.

Delayed parasite clearance during human stages of the malaria parasite life cycle due to reduced parasite sensitivity to antimalarial drugs increases the availability of gametocytes for mosquito stages of the cycle. Thus, IU in geographies with higher levels and trends of AMDR might evolve differently compared to those with less AMDR even with similar baseline *Pf*PR_2–10_, IU, and/or U5MR. This means that locations with similar *Pf*PR_2–10_ or IU in 2019 and U5MR in 2015 might have different malaria disease burden and child risk of death in 2023–2025 and/or subsequently if they have different AMDR profiles, thus warranting different vaccine allocation prioritization.

Furthermore, depending on their G_D_, D_D_, and I_M_, part of these AMDR-related new cases and their outcomes might not reach the health system, that is, G_D_, D_D_, and I_M_ impact vaccine needs, not only via VW but also by affecting the share and impact of community AMDR that is captured by the health system. Not only the outcomes and impacts but also the emergence and spread of antimicrobial-resistant mutant pathogens might be influenced dynamically by a multitude of factors. Among others, these include the following: (a) climate change [23]; (b) disasters (e.g., by compromising food security, then reducing immunity, then predisposing to infections and antimicrobial misuse, then increasing drug pressure); (c) migration (e.g., by transporting mutant gametocytes, then introducing mutant pathogens in other locations); and (d) drug pressure (e.g., due to poor prescribing practices, poor prescribing compliance, poor regulation). These factors, their potential interactions, and tempo-spatial dynamic impacts on malaria-related DALYs within and across generations might not be captured, at least not systematically and/or effectively, by past *Pf*PR_2–10_, malaria incidence, and/or U5MR based on a single-year each.

Prioritizing countries and subnational locations currently experiencing increasing levels of AMDR could maximize the effectiveness, equity, and impact of the vaccines. This could be accomplished by identifying/creating a summary measure to effectively capture not only static levels but also tempo-spatial trends of relevant molecular, clinical, and/or epidemiological features of AMDR of malaria parasites. This could then be fed into the vaccine allocation composite index. It could, therefore, be beneficial to revisit the composite index and potentially update it. It is particularly important to do so before the composite index is used on a larger scale for priority allocation of the currently more effective malaria vaccine R21.

Even *when* adequate vaccine doses reach high-risk countries, subnationally, children most-at-risk of malaria-related adverse health outcomes might not necessarily be prioritized in vaccination against the disease, that is, using a composite index that captures malaria transmission as well as system weaknesses and structural inequities should theoretically ensure at least in part that children most-at-risk of malaria-related adverse health outcomes receive the vaccine first. However, that might not be necessarily the case subnationally in most of the continent. This is because the same particularities that made these countries eligible for priority allocation of malaria vaccine may also make it more difficult to ensure within-country equitable prioritization and allocation. Traditionally, resources allocated to these countries do not reach or reach only partially or suboptimally those most in need due to rampant corruption and fragile institutions. SSA is the region with the highest level of corruption in the public sector [24]. And manifestations of corruption in the health sector include not only conditioning healthcare on informal payments from patients but also embezzlement and theft of medical products which then feed the private sector [25] whose access is limited to advantaged children. Why will the outcome be different when it comes to the malaria vaccine? The potential of malaria immunization to reduce inequity in access to existing interventions [26] can only be realized if the hurdles indicated here are addressed effectively. Otherwise, malaria vaccines may increase inequity.

Protecting malaria vaccines against hurdles faced by other vaccines and interventions subnationally across Africa is critical but challenging. To accomplish this, practical frameworks for within-country prioritization, allocation, and tracking are needed. These could include a subnational scoring system of all districts and/or municipalities per eligible country indicating the level of priority of each district and/or municipality to ensure that most-at-risk children are prioritized in malaria vaccination subnationally. To maximize equity in VN, different decision-making strategies may have to be considered depending on the parameters under consideration. For instance, to take VW into account in planning for equity, countries would first have to be ranked based on their proportion of low-VW subnational locations (pLVW). Here, using pLVW rather than VW is critical because the national average of VW rate does not capture within-country inequity. The ranking should be based on internationally established acceptable VW rate thresholds for each vaccine. Subsequently, using such a ranking, two strategies could be implemented for vaccine funds utilization. (i) Countries with high pLVW could be prioritized for vaccine delivery to maximize within-country efficiency. In these countries, within-country equity is already more likely given their higher pLVW. (ii) Countries with low pLVW could be prioritized for rigorous assessment and corrective programs to improve pLVW before vaccine delivery. For these countries, this strategy is more likely to address the root causes of not only low pLVW but also high and inequitable morbidity and mortality (see domain *Tackling core issues to maximize the impact of malaria immunization*). In the short term, the former and the latter might appear possibly less equitable (cross-country) and less efficient (within-country), respectively. However, this approach ensures the maximization of sustainable equity and equitable protection within (but also across) countries of both pLVW tiers. To facilitate real-time monitoring and evaluation by relevant stakeholders and the public, this should be coupled with robust and open data systems.

Putting in place these arrangements before deploying the vaccines is critical to ensure equitable within-country delivery and utilization of the vaccines to maximize child health protection. These subnational frameworks are necessary to strengthen the cross-country vaccine priority assessment tool created by the WHO. Otherwise, malaria vaccines could become just another tool to reinforce preexisting inequities on the continent, that is, without effective subnational allocation and tracking frameworks, the malaria vaccines could end up boosting the protection of low-risk children while leaving behind unprotected high-risk children, as it happens with other efforts by development partners despite their potential to contribute towards SDGs.

### Tackling core issues to maximize the impact of malaria immunization

**Fig. 2:**
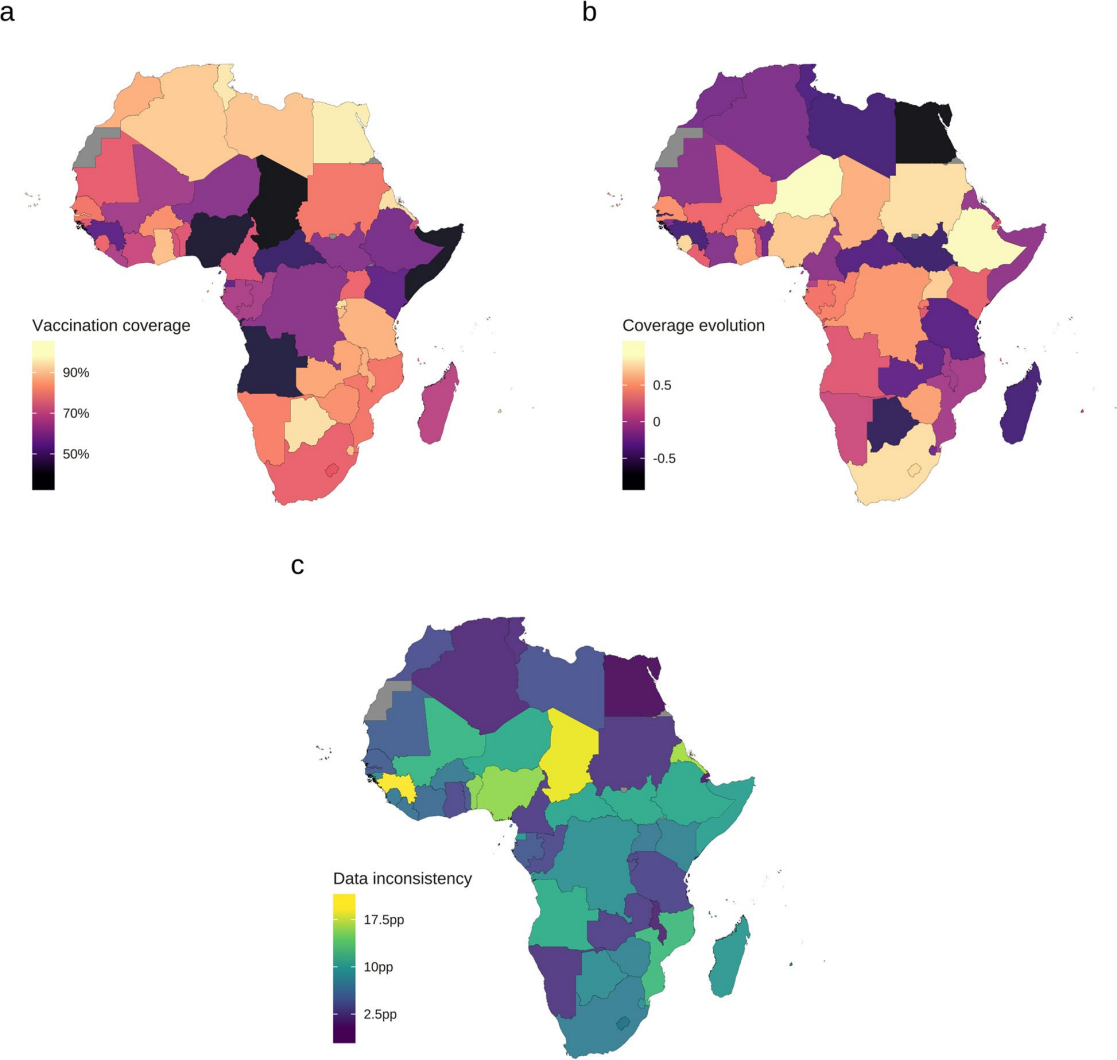
Summary of vaccination coverage and data inconsistency in national immunization in Africa, 2000–2022. **a** Overall vaccination coverage index. The color of the surface of each country denotes the national overall vaccination coverage index calculated by averaging the coverage indices across vaccines and then over the years, using the geometric mean. Thirty out of 54 countries had overall vaccination coverage < 80%. **b** Temporal evolution of national vaccination coverage. The color denotes the median Spearman’s *ρ* (*r*_s_). *r*_s_ > 0 indicates that overall national vaccination coverage (i.e., the geometric mean of vaccine-specific coverage indices) tends to increase over time and vice versa. Twenty-three countries had a negative evolution of overall vaccination coverage. **c** Vaccination coverage data inconsistency. The color denotes the average Euclidean distance (in percentage points) in coverage indices between different sources measuring the same quantity in the same country-year, averaged across vaccines and then over the years using the arithmetic mean. Fourteen countries had data inconsistency > 10 pp. For panels **a** and **b**: (i) the data covered the following vaccines (antigens): BCG (bacillus Calmette–Guérin); DTPCV1 (diphtheria-tetanus-pertussis-containing vaccine, 1st dose); DTPCV3; HEPB3 (HepB (hepatitis B), 3rd dose); HEPBBD (HepB, birth dose (given within 24 h of birth)); HIB3 (*Haemophilus influenzae *type b, 3rd dose); IPV1 (inactivated polio-containing vaccine, 1st dose); MCV1 (measles-containing vaccine, 1st dose); MCV2; PNCV3 (pneumococcal conjugate vaccine, final dose); POL3 (polio, 3rd dose); ROTAC (rotavirus, last dose); RCV1 (rubella-containing vaccine, 1st dose); and YFV (yellow fever vaccine); (ii) data from WUENIC (WHO/UNICEF Estimates of National Immunization Coverage) were used to derive the quantities shown. For panel **c**: (i) the data covered the vaccines (antigens) listed in the Supplementary material; (ii) data from the following sources were used for the calculations: administrative coverage, official coverage, WUENIC, PAB (protection at birth) estimates, and HPV (human papillomavirus) estimates. For all panels: multi-antigen vaccines were accounted for once per country-year in the calculations and COVID-19 vaccine was not included. Data source: [29]

Immunization against malaria will not be impervious to the chronic difficulties that traditionally reduce the value of public health interventions and programs in Africa. Indeed, little progress in core structural challenges [24, 27] has limited the impact of routine immunization on the continent. Several countries have remained off-track of global targets since the era of MDGs (see Fig. 2a, b). Other countries have reported progress but based on data with uncertain quality (see Fig. 2c). Overall, progress in routine immunization in Africa has been below international standards. SSA is the region with the highest prevalence of and inequalities (e.g., by residence, wealth, and mother’s education) in zero-dose (i.e., unvaccinated) and missed-dose (i.e., under-vaccinated) children globally [28], that is, countries where malaria vaccines will be rolled out are home to the largest share of children that EPIs fail to protect.

In addition to the limited supply of almost exclusively imported vaccines, compounded by VW, there are deep infrastructure weaknesses and governance issues that recurrently weaken health systems, thereby reducing the impact of immunization. VW is one of the manifestations of these core issues. It might have been more apparent at the height of the coronavirus disease 2019 pandemic. However, this typically underreported problem is far from new. Further compounding the difficulties are the rampant epidemics of corruption and mismanagement in most of the continent [24, 27]. These neglected epidemics further complicate the matters, among other pathways, by reducing the satisfaction and retention of the scanty healthcare workers (HCWs) and their capacity to perform professional tasks. As a result, most malaria-endemic countries have chronically understaffed health systems with overburdened HCWs and are increasingly facing brain drain and industrial action from HCWs. In the 47 countries of the AFR, there were, on average, only 1.55 doctors, nurses, and midwives per 1000 population in 2018 [30]. These preventable difficulties chronically impact negatively the performance of these countries in the provision of essential health services, including EPIs, with important consequences for population health.

Travel time to a healthcare facility in Africa, among the largest globally, is another major issue that creates persistent hurdles to childhood routine immunization. While mobile clinics have played a key role in providing access to vaccination for communities living in remote areas, scanty primary health care facilities have continued to be the main route for children to access EPI services. Thus, to get their children vaccinated, families have to travel long distances in settings without public transport. Effective and sustainable implementation of EPI is more challenging in such contexts. Consequently, outbreaks due to ancient, vaccine-preventable diseases—including measles and polio—have been increasing [31].

Despite the widely recognized value of evidence-based decision-making, little investment has been made into setting up and funding sustainable and open data systems across the continent, even around known major causes of death and suffering. Even the limited data that are collected are not used effectively internally. Typically, these are regarded only as a tool for external use to justify and/or request funding and not for the advancement of health governance and clinical practice nationally and subnationally. As a result, most problems contributing to the chronic inefficiency of public health interventions and programs in Africa are rarely understood and dealt with effectively and sustainably but impromptu. Limited availability and use of data contribute to limiting the progress toward eliminating vaccine-preventable diseases through various pathways. This includes reducing the effectiveness of policy-making and priority-setting, thereby reducing, e.g., the impact, equity, and sustainability of malaria vaccine allocation and utilization at all levels across and within countries. Given that, it can be seen why progress in malaria elimination has been meager in the AFR compared to other WHO regions [4], despite gains in disease control.

To ensure that malaria vaccines deliver their potential public health impact by protecting high-risk children, addressing these and other key issues must be an integral and central component of malaria immunization efforts and not an appendage. Addressing these chronic difficulties is inconceivable without compliance with the Abuja Declaration of 2001 and institutional strengthening. Thus, if the traditional *modus operandi* remains, these difficulties will impact malaria immunization equally, particularly given the need for four separate doses for full immunization, that is, adding all four doses of the vaccines to the current EPIs without drastic changes in the health systems may not benefit the children, at least not as expected. To fully and effectively address these chronic issues and realize the potential of malaria vaccines requires transformational change, which should be embedded in malaria immunization to effectively prevent past missteps.

## Conclusions

The temporal window to act effectively to transform our world towards a world free of malaria in the context of the SDGs is closing. To maximize the value of malaria vaccines to provide equitable protection to high-risk children, lessons learned from immunization against other vaccine-preventable diseases need to be embedded systematically in malaria immunization at all stages. Prevention of past missteps is a necessary condition if malaria vaccines are to become a transforming force in malaria control. Therefore, the rollout of malaria vaccines should be preceded by a continent-wide assessment program in order to identify disease-specific and system-wide hurdles and then implement adequate, context-specific corrective measures entangled with malaria vaccine deployment. Delivering the vaccines to high-risk countries without prior assessment and preparation might reduce their value and thus miss a unique opportunity to revert the recent setback and accelerate progress.

### Supplementary Information

**Supplementary Material 1.**

## Data Availability

The datasets used in the current study are publicly available and can be efficiently extracted from the sources cited in the article. The processed data that were presented in the main text and/or used to draw the graphs are available from the corresponding author upon reasonable request. The data processing and graph drawing were performed using R version 4.3.0. Data and code sharing will require a Materials Transfer Agreement (MTA).

## Abbreviations

AFR: WHO African Region
AMDR: Antimalarial drug resistance
CI: Confidence interval
D_D_: Developmental distance from MA
DALY: Disability-adjusted life year
DF: Number of indicated doses
EPI: Expanded Program on Immunization
G_D_: Geographical distance from MA
Gavi: Gavi, the Vaccine Alliance
GNI p.c.: Gross national income per capita
GTSM: Global Technical Strategy for Malaria
HCW: Healthcare worker
I_M_: Institutional mistrust
IU: Incidence among unvaccinated
MA: The most advantaged urban center
MDG: Millennium Development Goal
MVIP: Malaria Vaccine Implementation Programme
NNV: Number needed to vaccinate
*P. falciparum*: *Plasmodium falciparum*
*Pf*PR_2–10_: *P. falciparum* parasite prevalence rate in children aged 2–10 years
pLVW: Proportion of low-VW subnational locations
R21: R21/Matrix-M
R&D: Research and development
RTS,S: RTS,S/AS01
SDG: Sustainable Development Goal
SSA: Sub-Saharan Africa
U5: Children younger than 5 years
U5MR: All-cause under-5 mortality rate
VE: Vaccine efficacy
VN: Number of vaccines needed to prevent a malaria adverse outcome
VW: Vaccine wastage
WB: World Bank
WHO: World Health Organization
