# Supplementary material for: Malaria vaccination: hurdles to reach high-risk children
Source: BMC Med. 2024 Mar 13;22:111. doi: 10.1186/s12916-024-03321-2 (PMC10935779; doi:10.1186/s12916-024-03321-2)
Supplement: Supplementary file 1 — Supplementary Material 1. [file 12916_2024_3321_MOESM1_ESM.pdf]

Supplementary material for

Malaria vaccination: hurdles to reach high-risk children

Floriano Amimo, M.D., M.P.H., Ph.D. *[1]

[1]Faculty of Medicine, Eduardo Mondlane University, Maputo, Mozambique
*Correspondence: Faculty of Medicine, Eduardo Mondlane University, Maputo, Mozambique
Email: florianoamimo@gmail.com

Contents

Part 1: Vaccines (antigens) covered in the calculation of data inconsistency in vaccination coverage **1**

Part 1: Vaccines (antigens) covered in the calculation of data inconsistency in vaccination coverage

BCG: Bacillus Calmette–Guérin

DIPHCV4: Diphtheria-containing vaccine (DCV), 4th dose (1st booster)

DIPHCV5: DCV, 5th dose (2nd booster)

DIPHCV6: DCV, 6th dose (3rd booster)

DTPCV1: Diphtheria-tetanus-pertussis (DTP)-containing vaccine, 1st dose

DTPCV3: DTP-containing vaccine, 3rd dose

HEPB3: HepB (Hepatitis B), 3rd dose

HEPBBDALL: HepB, birth dose total (including those given within and after 24 hours of birth)

HEPBBD: HepB, birth dose (given within 24 hours of birth)

HIB3: *Haemophilus influenzae* type b, 3rd dose

HPVFEM: Human papillomavirus (HPV) female, final dose

HPVMALE: HPV male, final dose

IPV1FRAC: Fractional inactivated polio-containing vaccine (IPV), 1st dose

IPV1: IPV, 1st dose

IPV2FRAC: Fractional IPV, 2nd dose

IPV2: IPV, 2nd dose

MCV1: Measles-containing vaccine (MCV), 1st dose

MCV2: MCV, 2nd dose

MENACONJ: Meningococcal A conjugate vaccine

PABT: Protection at birth against neonatal tetanus

PNCV1: Pneumococcal conjugate vaccine (PNCV), 1st dose

PNCV2: PNCV, 2nd dose

PNCV3: PNCV, final dose

PERCV4: Pertussis-containing vaccine (PERCV), 4th dose (1st booster)

PERCVPW: PERCV, pregnant women

POL3: Polio, 3rd dose

RCV1: Rubella-containing vaccine, 1st dose

ROTA1: Rotavirus, 1st dose

ROTAC: Rotavirus, last dose

TT2PLUS: Tetanus toxoid-containing vaccine (TTCV), 2nd and subsequent doses

TTCV4: TTCV, 4th dose (1st booster)

TTCV5: TTCV, 5th dose (2nd booster)

TTCV6: TTCV, 6th dose (3rd booster)

TYPHOIDCONJ: Typhoid conjugate vaccine

YFV: Yellow fever vaccine
